# Effect of shRNA-mediated inhibition of Nanog gene expression on the behavior of human gastric cancer cells

**DOI:** 10.3892/ol.2013.1394

**Published:** 2013-06-13

**Authors:** WEN JI, ZHENG JIANG

**Affiliations:** Department of Gastroenterology, First Affiliated Hospital of Chongqing Medical University, YuZhong, Chongqing 400016, P.R. China

**Keywords:** Nanog, plasmid, SGC-7901, shRNA-Nanog, transfection

## Abstract

The aim of the present study was to employ RNA interference (RNAi) technology to construct and select shRNA-Nanog recombinant plasmids for the inhibition of Nanog gene expression and transfer these plasmids into the human gastric cancer cell line, SGC-7901, as well as to detect the expression of Nanog and the effects on the proliferation, migration, invasion, cell cycle and apoptosis of SGC-7901 cells. The pshRNA-Nanog interference plasmids were constructed and used to transfect SGC-7901 cells using lipofectamine. The expression of the Nanog gene was detected by fluorescence microscopy, RT-PCR and western blotting, and the most markedly inhibited group was identified. The SGC-7901 cells were transfected with recombinant shRNA-Nanog plasmids from the most markedly inhibited group using lipofectamine and the effect on proliferation was determined by CCK-8 assay. The migration and invasion of the SGC-7901 cells was determined by Transwell assays, while the cell cycle and apoptosis were analyzed by flow cytometry. The group with the highest inhibition rate was successfully constructed and identified. It was observed that the proliferation, invasion and migration capacity of the cells was reduced, that the cell cycle was arrested at the S phase and that apoptosis was significantly increased. The Nanog gene in gastric cancer cells is closely associated with cell proliferation, the cell cycle, apoptosis and migration and invasion abilities. The present study establishes the foundations for a novel approach for the genetic treatment of gastric cancer.

## Introduction

Gastric cancer mortality rates remain high despite advances in treatment. The theory that cancer stem cells play a role in the pathogenesis of gastric cancer (1) has opened up new avenues of diagnosis and treatment. The homeobox protein Nanog is a significant transcription factor in the maintenance of self-renewal and the pluripotency of embryonic stem cells (2–4). High expression levels of Nanog have been observed in embryonic germ cell tumors and certain somatic tumors (5). There are numerous studies demonstrating that Nanog is significant in the formation of solid tumors, as well as clinical data showing that the expression of Nanog protein in tumors is higher compared with corresponding adjacent tissues. The expression of Nanog is also closely correlated with the clinical classification of gastric carcinoma and the status and degree of lymph node saturation by the invasion of gastric cancer cells. Patients with an overexpression of Nanog have a poor prognosis (6). Chen *et al* reported that there may be gastric cancer stem cells in the gastric cancer tissues that express Nanog (7). These results demonstrated that the expression level of Nanog in gastric cancer tissues was higher compared with paracancerous tissues, and furthermore, that the expression of Nanog was correlated with tumor differentiation and malignancy. These findings also indicate the potential role of Nanog in the diagnosis and prognosis of gastric carcinoma. Cancer stem cells are responsible for the tumorigenicity of tumor cells and lead to tumor recurrence and metastasis (8). Gastric cancer stem cells have the ability to promote the formation of gastric cancer and maintain the self-renewal and constant proliferation of gastric cancer stem cells (9), suggesting that Nanog may be a new molecular marker for the diagnosis of gastric carcinoma. Previous studies have used qPCR to reveal that the expression levels of Nanog, Sox2, Lin28 and Oct-4 in tumor stem cells were different from other tumor cells and that the performance of miRNA inhibition technology in the two cell types also varied, suggesting that the two cell types had differing molecular mechanisms (10). Designing a specific miRNA method for cancer stem cells may be more specific and effective than current approaches (10). In the present study, RNA interference (RNAi) technology was used to inhibit the expression of the Nanog gene to study the effect on the tumor biological behavior of the gastric cancer cell line SGC-7901; the aim was to provide an experimental basis for the application of the RNAi technique as a gene therapy method for gastric cancer.

## Materials and methods

### Materials

The gastric cancer cell line, SGC-7901, *Escherichia coli* strain DH5α and plasmid pGenesil-1 were gifts from Dr Xiang Tingxiu (The First Affiliated Hospital of Chongqing Medical University, Chongqing, China). The annealing buffer contained 10 mM Tris (pH 8.0), 50 Mm NaCl and 1 Mm EDTA dissolved in 50 ml ddH_2_O, which was then filtered to remove bacteria and stored at 4°C. The RNAiso Plus, PrimeScript^®^ RT Reagent kit, Premix Taq® Version 2, restriction endonucleases *Bam*HI and *Hin*dIII and T4 DNA ligase were acquired from Takara (Otsu, Japan). The E.Z.N.A.^®^ Gel Extraction kit, E.Z.N.A Plasmid Mini kit I and E.Z.N.A. Endo-free Plasmid Mini kit I were purchased from Omega Bio-Tek (Norcross, GA, USA). Tryptone and yeast powder were acquired from Oxoid (Basingstoke, UK), while the RPMI 1640 was purchased from Hyclone (Logan, UT, USA) and the fetal bovine serum was from Hangzhou Sijiqing Biological Engineering Materials Co., Ltd. (Hangzhou, China). Trypsin, EDTA and the One-Step Competent Cell Preparation (SSCS) kit were purchased from Sangon Biotech Co., Ltd. (Shanghai, China) and lipofectamine 2000 was obtained from Invitrogen (Shanghai, China). The Nanog rabbit monoclonal antibody was supplied by Epitomics (Burlingame, CA, USA). The RIPA lysis buffer (strong), PMSF, SDS-PAGE Gel Preparation kit, BCA Protein Assay kit, BeyoECL Plus, HRP-labeled goat anti-rabbit IgG (H+L) and western primary antibody dilution solution were obtained from the Beyotime Institute of Biotechnology (Shanghai, China). Anti-β-actin was purchased from Beijing Biosynthesis Biotechnology Co., Ltd. (Beijing, China), while Cell Counting kit-8 was bought from Shanghai R&S Biotechnology Co., Ltd. (Shanghai, China), the Matrigel Basement Membrane Matrix was from BD Biosciences (San Jose, CA, USA) and the 24-well Millicell culture plates were from Milipore (Billerica, MA, USA).

### Design and synthesis of shRNA and PCR primers

First, the mRNA sequence of the human Nanog gene was identified in the NCBI gene bank. Next, according to the design principles of shRNA, three small interfering RNA (siRNA) targeting sequences with 21 bases were designed using online design software (http://zh.invitrogen.com/site/cn/zh/home/brands/ambion.html), as well as a 21-base unrelated sequence as a negative control. TTCG was used as a stem-loop to connect the siRNA target sequences and their reverse complementary sequences to form a hairpin structure, termed the shRNA. In addition, the transcriptional termination signal TTTTTA was added at the 3′-end. Finally the enzyme restriction sites were joined with *Bam*HI at the 5′-end and *Hin*dIII at the 3′-end. A BLAST search was performed with the sequences in the NCBI database to ensure the specificity of the three shRNA sequences (Table I). The three shRNA sequences and the negative control were sent to Invitrogen. According to the mRNA sequence of human Nanog in the NCBI GenBank (http://www.ncbi.nlm.nih.gov/nuccore/NM_024865.2), a pair of short fragment PCR primers were designed with the primer design software Primer Premier 5 and Oligo 6 and then synthesized by Takara (Table II).

### Construction of shRNA plasmid

The four pairs of oligonucleotide chains were separately dissolved in annealing solution, making the final concentration 0.5 g/l. A volume of 20 *μ*l of the up- and downstream primers for each pair was mixed, then incubated in a 95°C water bath for 5 min, followed by slow cooling to room temperature until annealing was completed. E.Z.N.A. Plasmid Mini kit I was used to extract the plasmid pGenesil-1 according to the manufacturer’s instructions. The pGenesil plasmids were cut by incubating them with *Bam*HI and *Hin*dIII in a 37°C water bath for 6 h, and then the samples were run on 1% agarose gel in 1X TAE. Subsequently, the E.Z.N.A. Gel Extraction kit was used to recycle restriction fragments according to the manufacturer’s instructions. Protein concentrations were measured with a protein nucleic acid analyzer (Thermo Fisher Scientific, Waltham, MA, USA), and T4 DNA ligase was used to connect the shRNA with the plasmid pGenesil-1 at 16°C overnight. The connected products were transformed into competent *Escherichia coli* DH5α, then bacteria solution was used to coat LB solid medium containing kanamycin (25 *μ*g/ml), which was incubated at 37°C for 16–20 h. Several monoclonal positive colonies were selected the next day and transferred into 4 ml LB liquid medium containing kanamycin (25 *μ*g/ml), which was placed in a 37°C, 200 rpm shaker to cultivate the bacteria for 12–16 h. E.Z.N.A. Plasmid Mini kit I was used to extract the recombinant plasmid, and enzyme identification and sequencing results demonstrated that the plasmids were correct, indicating that the four recombinant plasmids were successfully constructed. The four recombinant plasmids were named pshRNA-NanogA, pshRNA-NanogB, pshRNA-NanogC and pshRNA-negative control.

### Culture of SGC-7901 cells and transfection

A small bottle of SGC-7901 cell suspension was removed from a −80°C refrigerator, placed in a 37°C water bath and constantly agitated gently to ensure rapid thawing. The cell suspension was then transferred to a centrifuge tube, mixed with 2 ml RPMI 1640 medium containing 10% fetal bovine serum and centrifuged for 5 min at 800 × g. The supernatant was discarded. The cells were resuspended in 5 ml fresh culture medium containing 10% fetal bovine serum. The cells were transferred into a cell culture flask and cultured in an incubator at 37°C, with 5% CO2. The medium was changed once a day and when the SGC-7901 cells were at 80–90% confluence, the cells were digested with 0.25% trypsin solution containing 0.01% EDTA. Next, the cells were transferred into a centrifuge tube, centrifuged at 800 × g for 5 min and subcultured by 1:2 or 1:3, with approximately one passage every 2–3 days. The third or fourth generation were used for transfection. One day prior to transfection, the cells were transferred to a six-well plate, with ∼5×10^5^ cells per well, then stored in a 37°C, 5% CO_2_ incubator for one night. The endotoxin-free recombinant plasmids pshRNA-NanogA, pshRNA-NanogB, pshRNA-NanogC and pshRNA-negative control were extracted using the E.Z.N.A. Endo-Free Plasmid Mini Kit I according to the manufacturer’s instructions. When the SGC-7901 cells were at 80–90% confluence, the four groups of endotoxin-free recombinant plasmid were transfected into the cells in a liposome-mediated manner as follows: i) 4 g plasmid DNA was diluted in 250 *μ*l serum-free medium and gently mixed; ii) 10 *μ*l Lipofectamine 2000 was diluted in 250 *μ*l serum-free medium and incubated for 5 min at room temperature; iii) the diluted DNA was combined with diluted Lipofectamine 2000 (total volume, 500 *μ*l), then gently mixed and incubated for 20 min at room temperature; iv) 500 *μ*l complexes was added to each well containing cells and medium and mixed gently; and v) the cells were incubated at 37°C in a CO2 incubator. The medium was changed to be medium containing 10% fetal bovine serum after 4–6 h. The pGenesil-1-transfected and untreated SGC-7901 cells were used as control groups.

### Selection of the recombinant plasmid group with the highest inhibition rate

The green fluorescence (from plasmid penesil-1 with the enhanced green fluorescent protein (EGFP) label) of each of the groups was observed via inverted fluorescence microscopy at 48 h post-transfection. At 48 h post-transfection, RNAiso Plus was used to extract total RNA from each of the six groups of SGC-7901 cells, according to the manufacturer’s instructions. A protein nucleic acid analyzer was used to detect the purity and concentration of the RNA in each group, with an A_260_/A_280_ nm ratio of 1.80–2.0 meeting the purity requirements. mRNA to was converted to cDNA using the PrimeScript RT reagent kit; the reaction conditions were 37°C for 15 min, followed by 85°C for 5 sec. PCR was performed using Premix Taq Version 2.0, with Nanog gene primers P1 and P2 and β-actin primers P3 and P4. The reaction system used a 94°C initial denaturation for 5 min, a 94°C denaturation for 30 sec, a 53°C primer annealing for 30 sec, a 72°C extension for 35 sec for 25 cycles and a 72°C extension for 5 min. The products were run on a 2% agarose gel in 1X TAE, then images were captured with a Bio-Rad gel imaging instrument and the band intensity values were analyzed with Quantity One software.

Total protein was extracted from the cells of each group using RIPA lysis buffer and PMSF at a ratio of 100:1. Subsequent to protein denaturation, the protein samples were loaded and run using sodium dodecyl sulfonate-polyacrylamide gel electrophoresis (SDS-PAGE), followed by electrotransfer at 250 mA and membrane blocking with blocking buffer for 2 h. The first antibody, Nanog rabbit monoclonal antibody, was carefully added to the appropriate western primary antibody dilution solution (1:7,500), then incubated for one night at 4°C. The first antibody solution was poured off the membrane, which was then washed four times for 10 min with TBST buffer. The TBST buffer was then poured off and the secondary antibody, HRP-labeled goat anti-rabbit IgG (H+L), was added at the appropriate dilution in TBST buffer (1:1500), followed by incubation at 37°C for 1 h. The secondary antibody solution was then poured off the membrane, which was washed four times for 10 min with TBST buffer. In a dark room, BeyoECL Plus reagent was added to the PVDF membrane, which was rocked gently, while band development was observed. When the bands could be observed clearly, development was stopped by washing with distilled water for 30 min. Images of the bands on the membrane were captured with a Bio-Rad gel imaging instrument and the band intensity values were analyzed with Quantity One software.

### Effect of shRNA-NanogA on biological behavior of SGC-7901 cells

The recombinant plasmid with the highest inhibition rate was transfected into the SGC-7901 cells in a liposome-mediated, manner. There were four groups: The pshRNA-NanogA-transfected group, the pshRNA-negative control-transfected group, the pGenesil-1-transfected group and the normal SGC-7901 cell group. The effect on proliferation was determined by CCK-8, the cell cycle and apoptosis were analyzed via flow cytometry and the migration and invasion of the SGC-7901 cells was observed via Transwell tests.

### Detection of cell proliferation using CCK-8

Third or fourth generation cells were digested with 0.25% trypsin containing 0.01% EDTA, and the cells were transferred into a 96-well plate, with ∼6,000 cells per well. When the cells reached 70–80% confluence the next day, the four groups of endotoxin-free recombinant plasmid were transfected into the SGC-7901 cells in a liposome-mediated manner. The cells were then stored in a 37°C, 5% CO_2_ incubator. At 24, 48 and 72 h, 10 *μ*l CCK-8 solution was added to each well (starting volume of culture media, 100 *μ*l) so that the wells contained the same volume of culture medium and CCK-8 solution. Wells without cells were used as blank controls. The 96-well plate was then incubated for a further 2 h. A microplate reader was used to determine the absorbance of each group at 450 nm; three wells were used for each group to calculate the average absorbance value. Finally, cell proliferation curves were drawn to compare the cell proliferation of the groups.

### Detection of cell migration by Transwell assay

Four millicell inserts with 8-*μ*m diameter pores were placed into a 24-well plate. BD Matrigel and serum-free medium were mixed at a ratio of 1:9 and 100 *μ*l mixture was coated onto the upper surface of the millicells. Following incubation at 37°C for 4–5 h, the mixture solidified. The cells that had been transfected 24 h previously were digested, then 5×10^5^ cells were added into the upper compartment and RPMI 1640 medium containing 1% fetal bovine serum was used to supplement the cell suspension to 200 *μ*l. A total of 700 *μ*l RPMI 1640 medium containing 10% fetal bovine serum was added into the lower compartment and the 24-well plate was placed in a 37°C, 5% CO_2_ incubator for 20–24 h. The millicells were removed and gently washed twice with PBS, using cotton wool balls to wipe the cells on the upper surface. The cells were fixed with 4% paraformaldehyde for 20 min, then 0.1% crystal violet staining solution was added to stain the cells for 5–10 min, followed by two washes with PBS. When the cells were dry, the numbers of cells under the microporous membrane were counted using a microscope.

### Detection of cell invasion by Transwell assay

Four millicell inserts with 8-*μ*m diameter pores were placed into a 24-well plate. The cells that had been transfected 24 h previously were digested and 5×10^5^ cells were added into the upper compartment. RPMI 640 medium containing 1% fetal bovine serum was used to supplement the cell suspension to 200 *μ*l. A total of 700 *μ*l RPMI 1640 medium containing 10% fetal bovine serum was added into the lower compartment and the 24-well plate was placed in a 37°C, 5% CO_2_ incubator for 20–24 h. The millicells were removed and gently washed twice with PBS, using cotton wool balls to wipe the cells on the upper surface. The cells were fixed with 4% paraformaldehyde for 20 min, then 0.1% crystal violet staining solution was added to stain the cells for 5–10 min, followed by two more washes with PBS. When the cells were dry, the numbers of cells under the micro-porous membrane were counted using a microscope.

### Cell cycle analysis by flow cytometry

Third or fourth generation cells were inoculated into a six-well plate and incubated at 37°C, with 5% CO_2_ overnight. When the cells were at 70–80% confluence, they were transfected with the four groups of endotoxin-free recombinant plasmids in a liposome-mediated manner. Subsequent to 48 h, the cells were washed twice with pre-cooled PBS, digested with 0.25% trypsin containing 0.01% EDTA and transferred into EP tubes. The cells were then centrifuged for 5 min at 4°C using 8,000 × g and the supernatant was discarded. The cells were resuspended in 1 ml pre-cooled PBS, then centrifuged at 4°C using 8,000 × g for 5 min and the supernatant was discarded. The cells were fixed with 70% ice-cold ethanol and then incubated at 4°C for 24 h. The cell cycle of these cells was analyzed by flow cytometry the next day. Each experiment was repeated three times for each group and the results underwent statistical analysis.

### Detection of cell apoptosis by flow cytometry

Third or fourth generation cells were inoculated into a six-well plate and incubated at 37°C, with 5% CO_2_ overnight. When the cells were at 70–80% confluence, they were transfected with the four groups of endotoxin-free recombinant plasmids in a liposome-mediated manner. Subsequent to 48 h, the cells were washed twice with pre-cooled PBS, digested with 0.25% trypsin (without EDTA) and transferred into EP tubes. The cells were then centrifuged for 5 min at 4°C using 8,000 × g and the supernatant was discarded. The cells were resuspended in 1 ml pre-cooled PBS and apoptosis was detected by flow cytometry immediately. Each experiment was repeated three times for each group and the results underwent statistical analysis.

### Statistical analysis

Measurement data are expressed as the mean ± SD and were analyzed with a one-way ANOVA using SPSS 17.0 software (SPSS, Chicago, IL, USA). P<0.05 was considered to indicate a statistically significant difference.

## Results

### Identification of recombinant plasmid

**Figure 1.**
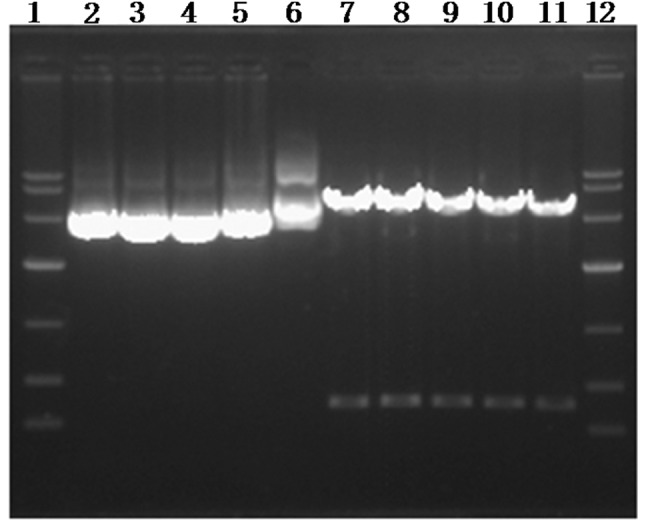
Identification of recombinant plasmid. A total of 12 lanes from left to right: 1,12, DNAMarker (DL 10000); 2, pshRNA-NanogA; 3, pshRNA-NanogB; 4, pshRNA-NanogC; 5, pshRNA-negative control; 6, pGenesil-1; 7, pshRNA-NanogA; 8, pshRNA-NanogB; 9, pshRNA-NanogC; 10, pshRNA-negative control; 11, pGenesil-1 digested by *Eco*RI and *Hind*III.

**Figure 2.**
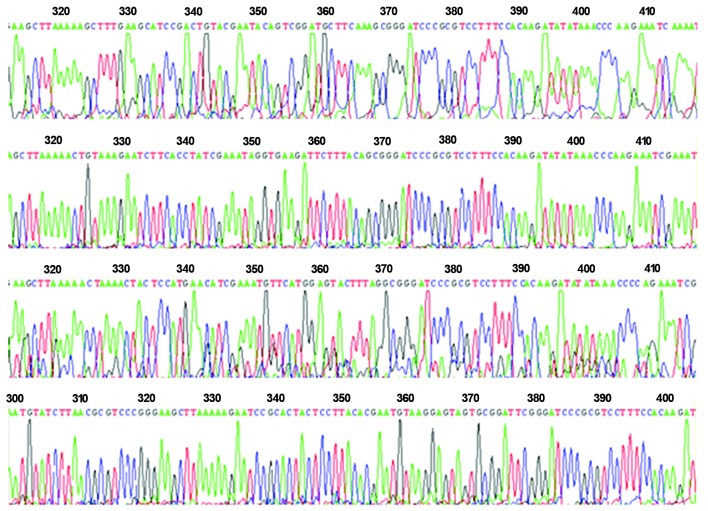
Sequencing chart. From top to bottom: pshRNA-NanogA, pshRNA-NanogB, pshRNA-NanogC and pshRNA-negative control.

The plasmids pshRNA-NanogA, pshRNA-NanogB, pshRNA-NanogC, pshRNA-negative control and pGenesil-1 were cut with *Bam*HI and *Hin*dIII in a 37°C water bath for 6 h. DNA fragments of 392 and 364 bp were cut in all groups (Fig. 1). Simultaneously, the sequencing results demonstrated the correct insertion of the shRNA oligonucleotide sequence (Fig. 2). This showed that the four recombinant plasmids were successfully constructed.

### Expression of green fluorescent protein

**Figure 3.**
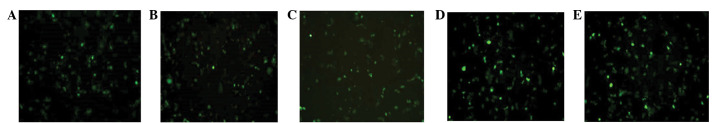
Fluorescence microscopy identification of green fluorescent protein expression [plasmid penesil-1 with an enhanced green fluorescent protein (EGFP) label] in SGC-7901 cells (magnification, x200) transfected with (A) pshRNA-NanogA; (B) pshRNA-NanogB; (C) pshRNA-NanogC; (D) pshRNA-negative control; and (E) pGenesil-1.

At 48 h post-transfection, the green fluorescence of each group was observed with an inverted fluorescence microscope. Green fluorescence was visible in the pshRNA-NanogA, pshRNA-NanogB, pshRNA-NanogC, negative control and pGenesil-1 plasmid groups. The green fluorescence intensities of the recombinant plasmid transfection groups were slightly weaker compared with the pGenesil-1 plasmid group and the transfection efficiency was ∼50%. There was no green fluorescence in the SGC-7901 cell group (Fig. 3).

### Nanog mRNA expression

**Figure 4.**
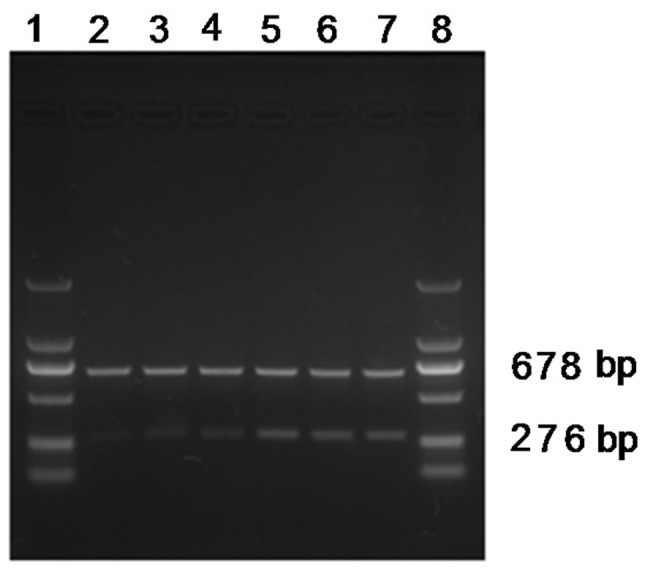
Effect of recombinant vector for Nanog mRNA in SGC-7901 cells as shown by semi-quantitative RT-PCR. From left to right: 1,8, DNA Marker (DL 2000); 2, transfected with pshRNA-NanogA; 3, transfected with pshRNA-NanogB; 4, transfected with pshRNA-NanogC; 5, transfected with pshRNA-negative control; 6, transfected with pGenesil-1; 7, SGC-7901.

At 48 h post-transfection, total RNA was extracted from each of the six groups of SGC-7901 cells. qPCR was performed according to the previously described system and conditions and β-actin was used as an internal reference. All the β-actin (678 bp) groups were of consistent brightness. The Nanog staining in the pshRNA-NanogA-transfection group (276 bp) was the faintest and the pshRNA-NanogB and pshRNA-NanogC groups were the second faintest, while the phRNA-negative control, pGenesil-1 and SGC-7901 cell groups were of the same brightness (Fig. 4). The analysis of the band intensities using Quantity One software showed that the Nanog mRNA expression of the pshRNA-NanogA group was 14.03±0.37%, while that of the pshRNA-NanogB group was 21.29±1.66%, the pshRNA-NanogC group was 21.26±0.94%, the pshRNA-negative control was 43.92±6.29%, the pGenesil-1 group was 42.88±2.83% and the SGC-7901 cell group was 46.77±6.72%. There were significant differences between the recombinant plasmid groups (the pshRNA-NanogA, pshRNA-NanogB and pshRNA-NanogC groups) and the other groups (the pshRNA-negative control, pGenesil-1 and SGC-7901 cell groups; P<0.05). The pshRNA-NanogA group was significantly different from the pshRNA-NanogB and pshRNA-NanogC groups (P<0.05), while no significant differences were observed between the pshRNA-NanogB and pshRNA-NanogC groups (P>0.05), as well as among the pshRNA-negative control, pGenesil-1 and SGC-7901 cell groups (P>0.05). The results showed that the pshRNA-NanogA, pshRNA-NanogB and pshRNA-NanogC groups all had inhibitory effects on the expression of Nanog mRNA, and that the inhibitory effect of pshRNA-NanogA was more evident.

### Effect on Nanog protein expression

**Figure 5.**
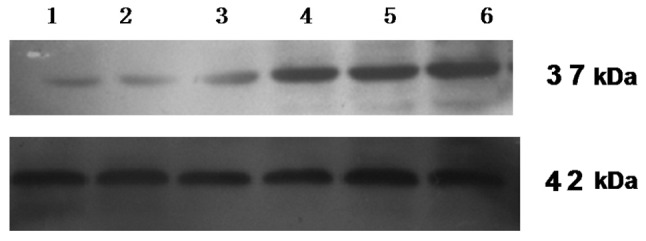
Effect of recombinant vector for Nanog protein in SGC-7901 cells as shown by western blotting. From left to right: 1, transfected with pshRNA-NanogA; 2, transfected with pshRNA-NanogB; 3, transfected with pshRNA-NanogC; 4, transfected with pshRNA-Negative control; 5, transfected with pGenesil-1; 6, SGC-7901. The top image shows a 37 kDa Nanog protein and bottom image shows a 42 kDa β-actin protein.

Human Nanog has 305 amino acids encoding a 37 kDa protein, while the β-actin protein is 42 kDa. At 48 h post-transfection, total protein was extracted from each of the six groups of SGC-7901 cells. A western blot analysis was performed to detect Nanog and β-actin protein expression. The results showed that the β-actin of all the groups had the same brightness. The Nanog expression of the pshRNA-NanogA transfection group was the faintest and the pshRNA-NanogB and pshRNA-NanogC groups were the second faintest, while the phRNA-negative control, pGenesil-1 and SGC-7901 groups were of the same brightness (Fig. 5). The analysis of the band intensities using Quantity One Quantity One software showed that the Nanog protein expression of the pshRNA-NanogA group was 61.57±0.81%, the pshRNA-NanogB group was 77.32±0.61%, the pshRNA-NanogC group was 83.62±8.32%, the pshRNA-negative control group was 94.60±1.47%, the pGenesil-1 group was 90.40±3.16% and the SGC-7901 cell group was 93.32±1.88%. There were significant differences between the recombinant plasmids groups (pshRNA-NanogA, pshRNA-NanogB and pshRNA-NanogC groups) and the other groups (pshRNA-negative control, pGenesil-1 and SGC-7901 groups; P<0.05). The pshRNA-NanogA group was significantly different from the pshRNA-NanogB and pshRNA-NanogC groups (P<0.05), while no significant differences were observed between the pshRNA-NanogB and pshRNA-NanogC groups (P>0.05), as well as among the pshRNA-negative control, pGenesil-1 and SGC-7901 cell groups (P>0.05). The results showed that the pshRNA-NanogA, pshRNA-NanogB and pshRNA-NanogC groups all had inhibitory effects on the expression of Nanog protein. The inhibitory effect of pshRNA-NanogA was the greatest. This result was consistent with the RT-PCR results, so recombinant plasmid pshRNA-NanogA was the most suitable choice for the following experiments.

### Nanog shRNA-transduced cells exhibit decreased proliferation

**Figure 6.**
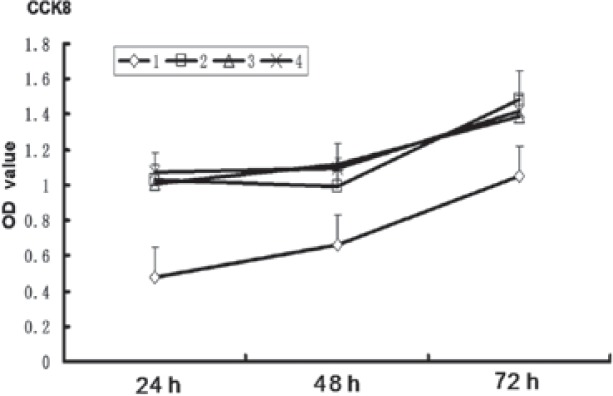
Effect of recombinant plasmids on SGC-7901 cell growth in: 1, pshRNA-Nanog group cells, 2, negative control group cells: 3, pGenesil-1 group cells, 4; SGC-7901 group cells.

The cell proliferation curve (Fig. 6) showed that, compared with the pshRNA-negative control, pGenesil-1 and SGC-7901 cell groups, the proliferative ability of the pshRNA-NanogA group was significantly restricted (P<0.05). No significant differences were observed among the pshRNA-negative control, pGenesil-1 and SGC-7901 cell groups (P>0.05).

### shRNA-Nanog inhibits the tumor cell migration capacity

**Figure 7.**
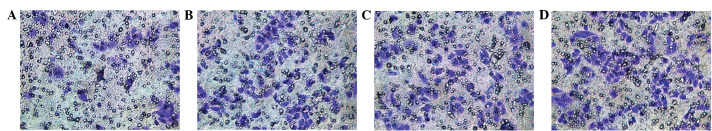
Cell migration assay. (A) pshRNA-Nanog group cells, (B) negative control group cells, (C) pGenesil-1 group cells and (D) SGC-7901 group cells. Cells were stained by 0.1% crystal violet staining solution. Coloring indicates transmembrane cells.

At 24 h after the transfection of the cells in the upper compartments of the millicells (Fig. 7), all the groups displayed cells that had crossed the Matrigel-coated membranes. The number of cells that crossed the membrane in the pshRNA-NanogA group (37.55±1.83) was less than that of the pshRNA-negative control (84.21±1.71), pGenesil-1 (86.23±2.31) and SGC-7901 cell (89.40±3.98) groups, and the differences were significant (P<0.05). No significant differences were observed among the pshRNA-negative control, pGenesil-1 and SGC-7901 cell groups (P>0.05).

### shRNA-Nanog inhibits the tumor cell invasion capacity

**Figure 8.**
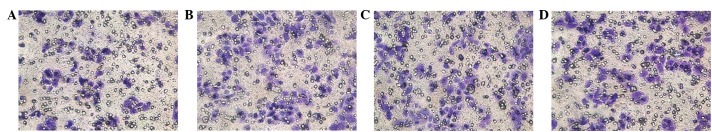
Cell invasion assay. (A) pshRNA-Nanog group cells, (B) the negative control group cells, (C): pGenesil-1 group cells and (D) SGC-7901 group cells. Cells were stained by 0.1% crystal violet staining solution. Coloring indicates transmembrane cells.

At 24 h after the transfection of the cells in the upper compartments of the millicells (Fig. 8), all the groups exhibited cells that had crossed the membranes. The number of cells that crossed the membrane in the pshRNA-NanogA group (41.23±2.76) was less than that of the pshRNA-negative control (84.11±2.37), pGenesil-1 (90.71±2.78) and SGC-7901 cell (86.00±3.24) groups, and the differences were significant (P<0.05). No significant differences were observed among the pshRNA-negative control, pGenesil-1 and SGC-7901 cell groups (P>0.05).

### Cell cycle progression is blocked in Nanog shRNA-transduced cells

**Figure 9.**
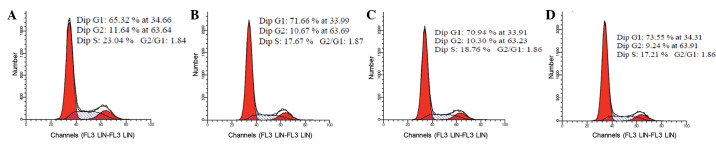
Cell cycle distribution of SGC-7901 cell by flow cytometry. (A) pshRNA-NanogA group cells, (B) negative control group cells, (C) pGenesil-1 group cells, (D) SGC-7901 group cells.

At 48 h post-transfection, the cell cycle distributions of these cells were detected by flow cytometry (Fig. 9). Compared with the pshRNA-negative control, pGenesil-1 and SGC-7901 cell groups, there were more S-phase cells in the pshRNA-NanogA group (P<0.05). No significant differences were observed among the pshRNA-negative control, pGenesil-1 and SGC-7901 cell groups (P>0.05).

### RNAi-mediated Nanog knockdown leads to cell apoptosis

**Figure 10.**
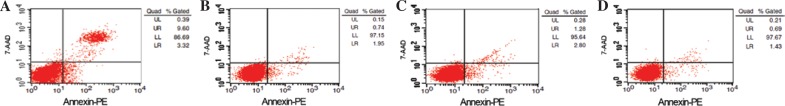
Apoptosis analysis of SGC-7901 cell by flow cytometry. (A) pshRNA-NanogA group cells, (B) negative control group cells, (C) pGenesil-1 group cells, (D) SGC-7901 group cells.

At 48 h post-transfection, the cell apoptosis of these cells was detected by flow cytometry (Fig. 10). Compared with the pshRNA-negative control, pGenesil-1 and SGC-7901 cell groups, the number of apoptotic cells in the pshRNA-NanogA group was significantly increased (P<0.05). No significant differences were observed among the pshRNA-negative control, pGenesil-1 and SGC-7901 cell groups (P>0.05).

## Discussion

Research has shown that Nanog, a transcription factor expressed in primordial germ cells and embryonic stem cells, is an important regulatory factor for maintaining the self-renewal and pluripotency of gastric cancer stem cells (9). Since Nanog is involved and important in the occurrence and development of gastric cancer, gene-targeted therapy for Nanog may become an important method for treating gastric cancer.

RNAi technology is a popular bio-technology that is highly specific, works quickly and has a high efficiency and low cost. The mechanism behind RNAi is post-transcriptional double-stranded RNA (dsRNA)-mediated gene silencing to induce specific target gene mRNA degradation (11,12), thus inhibiting protein synthesis to affect target gene function. In the present study, a human Nanog gene interference plasmid, pshRNA-Nanog was successfully constructed and transfected into the gastric cancer cell line, SGC-7901. Green fluorescent protein was then observed by fluorescence microscopy and recombinant plasmids were successfully transfected into the cells with a transfection efficiency of ∼50%. Nanog expression at the mRNA level was detected through RT-PCR, while Nanog protein expression was detected with western blotting. Nanog gene expression was observed to have dropped significantly in these experiments. The group of recombinant plasmids with the highest inhibition rate was selected and transfected into the SGC-7901 cells with the result being that the proliferative ability of the pshRNA-NanogA group was significantly restricted. The cell cycle of the Nanog shRNA-transduced cells was blocked in the S-phase, showing that DNA synthesis was blocked, while the ability for cell proliferation was inhibited. Apoptotic cells counts in the pshRNA-NanogA group significantly increased, indicating that the recombinant plasmid of pshRNA-NanogA inhibited Nanog gene expression and was involved in inducing cell apoptosis. However, malignant tumor treatment is not only for the control of the primary lesion; invasion and metastasis form the malignant phenotype of tumors, and blocking tumor metastasis is likely to be more effective. Transwell and Matrigel are used to simulate the body environment and have been widely used in the study of tumor migration and invasion (13). In the present Transwell invasion and migration assays, the number of cells that crossed the membrane in the shRNA-Nanog-inhibited group was significantly reduced. This indicated that inhibiting Nanog gene expression in gastric cancer cells may inhibit the invasion and migration of human gastric cancer cells.

RNAi technology was able to inhibit the expression of Nanog, thereby inhibiting tumor cell proliferation, migration and invasion. This method may provide an experimental basis for a gene therapy approach for treating gastric cancer.

## Figures and Tables

**Table I.** Design of shRNA.

Target sequences	Nucleotide sequences
1. GCTTTGAAGCATCCGACTGTA	5′-GATCCCGCTTTGAAGCATCCGACTGTATTCGTACAGTCGGATGCTTCAAAGCTTTTTA-3′
	5′-AGCTTAAAAAGCTTTGAAGCATCCGACTGTACGAATACAGTCGGATGCTTCAAAGCGG-3′
2. CTGTAAAGAATCTTCACCTAT	5′-GATCCCGCTGTAAAGAATCTTCACCTATTTCGATAGGTGAAGATTCTTTACAGTTTTTA-3′
	5′-AGCTTAAAAACTGTAAAGAATCTTCACCTATCGAAATAGGTGAAGATTCTTTACAGCGG-3′
3. CCTAAACTACTCCATGAACAT	5′-GATCCCGCCTAAACTACTCCATGAACATTTCGATGTTCATGGAGTAGTTTAGGTTTTTA-3′
	5′-AGCTTAAAAACCTAAACTACTCCATGAACATCGAAATGTTCATGGAGTAGTTTAGGCGG-3′
4. GAATCCGCACTACTCCTTACA (The negative control)	5′-GATCCGAATCCGCACTACTCCTTACATTCGTGTAAGGAGTAGTGCGGATTCTTTTTTA-3′
	5′-AGCTTAAAAAAGAATCCGCACTACTCCTTACACGAATGTAAGG AGTAGTGCGGATTCG-3′

**Table II.** PCR primers.

Gene	Primer sequence	Product size (bp)
Nanog	P1: 5′-ACTGTCTCTCCTCTTCCTTCCTC-3′	
	P2: 5′-GGTCTTCACCTGTTTGTAGCTG-3′	276
β-actin	P3: 5′-ACTGTGCCCATCTACGAGG-3′	
	P4: 5′-GAAAGGGTGTAACGCAACTA-3	678
